# Age of Information Minimization for Radio Frequency Energy-Harvesting Cognitive Radio Networks

**DOI:** 10.3390/e24050596

**Published:** 2022-04-24

**Authors:** Juan Sun, Shubin Zhang, Changsong Yang, Liang Huang

**Affiliations:** 1 College of Computer Science and Technology, Zhejiang University of Technology, Hangzhou 310014, China; sunjuan@zjut.edu.cn (J.S.); zhangshubin@zjut.edu.cn (S.Z.); 2 Guangxi Key Laboratory of Cryptography and Information Security, Guilin University of Electronic Technology, Guilin 541004, China; csyang@guet.edu.cn

**Keywords:** Age of Information, RF energy-harvesting, cognitive radio network, dynamic programming

## Abstract

The Age of Information (AoI) measures the freshness of information and is a critic performance metric for time-sensitive applications. In this paper, we consider a radio frequency energy-harvesting cognitive radio network, where the secondary user harvests energy from the primary users’ transmissions and opportunistically accesses the primary users’ licensed spectrum to deliver the status-update data pack. We aim to minimize the AoI subject to the energy causality and spectrum constraints by optimizing the sensing and update decisions. We formulate the AoI minimization problem as a partially observable Markov decision process and solve it via dynamic programming. Simulation results verify that our proposed policy is significantly superior to the myopic policy under different parameter settings.

## 1. Introduction

To cope with both the spectrum scarcity and the energy shortage challenges in future wireless networks, radio frequency (RF) energy-harvesting in cognitive radio networks (CRN) has been increasingly attractive. Cognitive radio technology allows secondary users (SUs) to opportunistically access the primary users’ (PUs) licensed spectrum, based on the condition that the SUs transmission must not cause harmful interference to PUs [1,2,3,4]. Meanwhile, the RF energy-harvesting technique conquers the intermittency and uncontrollability of the conventional charging techniques absorbing energy from renewable energy sources [5,6,7]. Hence, it can simultaneously improve energy efficiency and spectral efficiency, where the SUs can both capture energy and spectrum [8].

While existing works mainly investigated throughput of the RF energy-harvesting CRN, many emerging applications require timely status-update delivery [9,10,11,12,13,14,15], i.e., health monitoring, environment monitoring, smart building, vehicle-to-vehicle networking, and so on. For example, in health monitoring, the sensors continuously measure and update blood pressure and heartbeat to the health monitoring platform, which implies the importance of the freshness and timeliness of status-update. The Age of Information (AoI) as a recently proposed performance metric can be used to quantify the freshness and timeliness of status-update [16,17,18,19,20,21,22,23]. It is defined as the time elapsed since the generation time of the latest successfully received status-update at the destination.

Some innovative efforts have been devoted to the AoI of CRN [24,25,26,27,28]. In [24], the authors considered a cognitive wireless sensor network with a cluster of SUs, where the authors proposed a joint and scheduling strategy that optimized energy efficiency of a communication system subject to the expected AoI. The authors in [25] considered an overlay CRN where the SU acted as a relay. The SU forwarded the PU’s packets or transmitted its own packets. The optimal policy for status-update and packet relaying was investigated to minimize the average AoI and energy efficiency. In [26], the authors analyzed the average peak AoI of the PU and SU for both overlay and underlay schemes. The asymptotic expressions of the average peak AoI were derived when the PU operated at high signal-to-noise ratio. Considering that it is difficult for PU keeping time-slotted synchronization with SU, the authors in [27] investigated AoI minimization in CRN with an unslotted PU. The closed-form expression was derived by conducting a Markov chain analysis. In [28], the authors considered AoI minimization for energy-harvesting CRN. They assumed that the SU harvests energy from ambient energy sources and derived the optimal sensing and update policies for both perfect and imperfect spectrum sensing.

Overall, the aforementioned research efforts rarely address AoI minimization for RF energy-harvesting CRN. Motivated by this, this article attempts to minimize the average AoI by adaptively making sensing and updating decisions subject to the energy causality and spectrum constraints with imperfect spectrum sensing. The system consists of one PU and one SU. Different from [28], the SU harvests RF energy from PU transmissions instead of ambient energy sources, which is further used to generate and deliver the status-update data pack when the PU is idle. The SU utilizes the harvested energy to perform spectrum sensing and updating. The main contributions of this paper are summarized as follows:

- We study the average AoI minimization for RF energy-harvesting CRN where the SU harvests energy from PU transmissions. In each time slot, the SU adaptively makes sensing and updating decisions based on the channel state information, the AoI value, the available energy, and the belief of PU’s spectrum.
- We formulate the decision-making problem as a framework of a partially observable Markov decision process (POMDP) with finite state and action spaces. Then we use dynamic programming to obtain the optimal policy.
- We demonstrate through extensive simulations that the proposed policy can essentially improve the system performance compared to the myopic policy under different system parameter settings.

The remaining part of the paper is organized as follows. In Section 2, we review the works on RF energy-harvesting CRN in the literature. Section 3 describes the studied system model for RF energy-harvesting CRN. Section 4 first formulates the AoI minimization problem as a POMDP framework and then solves it through the dynamic programming. Section 5 presents simulation results and discussions. Finally, Section 6 concludes this paper.

## 2. Related Works on RF Energy-Harvesting CRN

Recently, cognitive radio technology has drawn significant attention as a promising solution to overcome the licensed spectrum severe scarcity. Cognitive radio allows SUs to opportunistically access PUs’ licensed spectrum, based on the condition that the SUs transmission must not cause harmful interference to PUs [1,2,3]. Spectrum sensing is an important functionality in the cognitive radio system [29], by which the SUs decide whether the spectrum is occupied by the PUs. It can be performed by a single SU or in cooperation with multiple SUs. The SUs can only transmit data when the PUs are idle [30]. Various spectrum-sensing approaches have been developed based on employing different features of the PUs’ signal [31], such as coherent detection [32], energy detection [33], and feature detection [34].

On the other hand, energy shortage is also a challenge in future wireless networks. Over the last past years, the RF energy-harvesting technique has emerged as a candidate method for charging low-power wireless devices, which can conquer the intermittency and uncontrollability of the conventional charging techniques absorbing energy from renewable energy sources [5,6,7]. In [35], the authors proposed the harvest-then-transmit (HTT) protocol as one of the important transmission strategies of RF energy-harvesting technology, where the users first harvest energy from the hybrid access point (HAP) and then use the captured energy to transmit information to the HAP. There have been some related works before. In [36], the authors investigated the wireless-powered network (WPCN) where one HAP coordinated the wireless information and energy transmissions to a set of nodes, where the transmission completion time minimization subject to the throughput requirement per node was considered. Furthermore, the authors studied a similar WPCN scenario in [37], where they focused on energy provision minimization for two physical-layer protocols, non-orthogonal multiple access (NOMA) and time-division multiple access (TDMA). Different from the common WPCN with a fixed HAP, the transmission completion time minimization was investigated in aerial vehicle-enabled WPCN in [38].

To jointly solve the aforementioned two challenges including spectrum scarcity and energy shortage, introducing RF energy-harvesting in CRN has been increasingly attractive due to the fact that it can simultaneously improve energy efficiency and spectral efficiency, where the SUs can both capture energy and spectrum [8]. The timely-delivery probability of data packs for the RF energy-harvesting SU was derived in [39], where the SU opportunistically accesses the spectrum vacated by the PU to deliver real-time data packs and harvests RF energy when the PU is active. Unlike the traditional RF energy-harvesting CRN system where the SU keeps synchronization with the PU, the authors in [40] considered unslotted PU. The sensing intervals were derived to balance between energy harvesting and spectrum access. However, both [39,40] focused on a simple CRN consisting of one PU and one SU. The authors in [41,42,43] considered a more general scenario where there were multiple SUs or multiple PUs. In [41], the multiple selection strategy was proposed for RF energy-harvesting CRN to maximize the SUs’ average throughput. In [42], the authors studied a hybrid energy-harvesting SU that can capture energy from both renewable sources and ambient radio frequency signals. The asymptotic activity behavior of a single SU was analyzed by deriving the theoretical upper bound on sensing and transmission opportunities. In [43], the authors investigated the end-to-end throughput maximization by jointly optimizing the transmit power and time allocation for multiple SUs.

## 3. System Model

**Figure 1:**
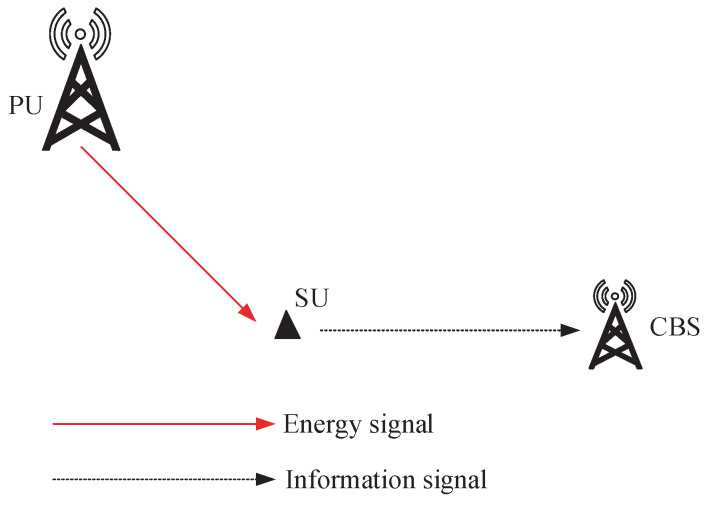
System model. In each time slot, the SU can harvest energy from the PU transmissions and can deliver the status-update date pack to the CBS when the channel is idle.

As illustrated in Figure 1, we investigated AoI minimization for a RF energy-harvesting CRN, where the system consists of one PU, one SU, and one CBS communicating with the SU. The SU is a wireless sensor node that monitors the physical process and randomly generates status updates to the CBS. It has no embedded power supply available and harvests RF energy from PU transmissions. Additionally, it opportunistically accesses the PU’s licensed spectrum. We considered a time-slotted system with a time interval of *T* time slots. The duration of each time slot is sufficient for the SU to generate one status-update data packet and receive it successfully at the CBS. Without loss of generality, we assume that the time slot duration is 1 s. The important notations are summarized in Table 1.

### 3.1. Primary User Model

The occupancy of a channel by the PU is modeled as a two-state continuous-time Markov chain [44], i.e., active (*A*) and idle (*I*) states. In each time slot, the PU either stays in the idle state or occupies the spectrum in an active state. The two-state (active/idle) Markov chain model for modeling PU activity has been verified to be an appropriate model to characterize spectrum occupancy in the time domain [45]. Let $q_{t}\in \left\{A,I\right\}$ denote the state of the PU for t=0,1,…,T−1. The transition probabilities of the two-state Markov chain are expressed as $p_{ai}$ and $p_{ii}$, which represent transitioning from the active state to the idle state, and still staying in the idle state, respectively. For t=0,1,…,T−1, we have

(1) $$p_{ai}≜P\left(q_{t+1}=I|q_{t}=A\right),$$

(2) $$p_{ii}≜P\left(q_{t+1}=I|q_{t}=I\right).$$

The transition probabilities are known to SU, which can be obtained by long-term measurements.

### 3.2. Secondary User Model

We considered the SU time-slotted synchronization with the PU. At the beginning of each time slot, the SU needs to decide whether to sense the PU’s spectrum. If it decides not to sense the spectrum, it takes the entire time slot to harvest energy from the PU transmissions. That is, energy can be harvested when the PU is active; otherwise, no energy is harvested. We assume the imperfect sensing outcome for the SU [46]. We denote the probability of a false alarm by $p_{f}$ (i.e., the probability of deciding the spectrum is occupied by the PU while it is not). The probability of detection is denoted by $p_{d}$ (i.e., the probability of deciding PU is active when it is active). Then, we have

(3) $$p_{f}=P\left(\hat{q_{t}}=A|q_{t}=I\right),$$

(4) $$p_{d}=P\left(\hat{q_{t}}=I|q_{t}=I\right).$$

The SU will take two actions after obtaining the sensing result. When the PU is sensed to be active, the SU will not deliver the status-update data pack. This means that it can harvest energy when the PU is actually active. On the other hand, if the sensing result is that the spectrum is vacated by the PU, the SU needs to further decide whether to update. If an update package is delivered, the SU will receive a 1-bit feedback signal from the CBS to determine whether the update is successful or not. When the sensing result $\hat{q_{t}}=I$ is correct, the update is successful. This happens with probability $1-p_{f}$. Update failure occurs if the PU is active despite the SU declaring it idle. This happens with probability $1-p_{d}$. The SU aims to minimize the average AoI by making the optimal sensing and update decisions over time slot t=0,1,…,T−1. We denote the decision of time slot *t* by $x_{t}=\left(\phi_{t},\theta_{t}\right)$, where $\phi_{t}$∈{0(notsense),1(sense)} and $\theta_{t}\in \left\{0\left(\mathrm{not}\;\mathrm{update}\right),1\left(\mathrm{update}\right)\right\}$ denote the sensing and update decisions, respectively. The optimal sensing and update decisions are based on the SU’s states and its statistical knowledge of the PU activity.

(1) Belief model: The SU observes the availability of the PU spectrum by adaptively detecting and accessing the spectrum. The belief state of the PU spectrum can be obtained based on the SU’s action and observation history. That is, at the beginning of each time slot *t*, the SU forms the belief $\rho_{t}$. The belief $\rho_{t}$ is the conditional probability that the PU is in an idle state given the SU’s action and observation history.

(2) Channel model: Denote the channel power gains from the PU to the SU and from the SU to the CBS by $h_{t}$ and $g_{t}$ over time slot *t*. We consider a quasi-static channel model based on one time slot by assuming that the channel state information is constant in a single time slot and variable in different time slots. Especially, as is commonly assumed in the works about the wireless communication system, the channel state information of the current time slot can be perfectly obtained.

(3) RF energy-harvesting model: The batter-free SU harvests energy from the occupied spectrum by the PU. For the SU, the HTT protocol is employed. That is, the SU first captures energy from the PU transmissions and then utilizes the harvested energy to sense spectrum and transmit data. Overall, there are two cases where energy can be harvested over time slots: (1) The not sensing decision is made, and the PU is inactive, and (2) the sensing decision is made, and the sensing result $\hat{q_{t}}=A$ is correct. The energy captured by the SU is expressed as

(5) $$E_{H,t}^{m}=\eta \tau Ph_{t},$$

for t=0,1,…,T−1 and m=1,2, where η, τ and *P* denote the energy-harvesting efficiency, energy-harvesting time and transmit power at the PU, respectively. The superscript *m* denotes the two cases of energy-harvesting mentioned above. The captured energy is used to perform sensing spectrum and update. Denote the energy and time consumption on sensing spectrum by δ and $\tau_{s}$, respectively. Similarly, let $E_{T,t}$ and $\tau_{t}$ denote the energy and time consumption on update, respectively. Energy consumption $E_{T,t}$ is time-varying, which is related to the channel state information $g_{t}$ from the SU to the CBS. According to Shannon’s formula [47], the transmission rate $\frac{S}{\tau_{t}}$ can be expressed as $\frac{S}{\tau_{t}}=W\log_{2}\left(1+\frac{E_{T,t}g_{t}}{\tau_{t}\sigma^{2}}\right)$, where $\sigma^{2}$ is the noise power at the CBS, *S* is the size of status-update data pack, and *W* is the bandwidth. Reorganizing the expression, we obtain the energy consumption, $E_{T,t}$, as

(6) $$E_{T,t}=\frac{\sigma^{2}\tau_{t}}{g_{t}}\left(2^{\frac{S}{\tau_{t}W}}-1\right).$$

Since the size of the status-update data pack is fixed, $E_{T,t}$ is only related to the channel state information from the SU to the CBS. Although the update decision can reduce the AoI to one, when the channel quality is poor, it may be better not to deliver the status-update data pack to conserve energy. Note that update failure occurs if the sensing result $\hat{q_{t}}=I$ is incorrect. In this case, the SU will consume all its available energy. Let $B_{\max}$ denote the battery capacity of the SU. In time slot *t*, the battery state is $b_{t}$, which evolves as

(7) $$b_{t+1}=\min\left\{b_{t}+E_{H,t}^{m}-\phi_{t}\delta -\theta_{t}E_{T,t},B_{\max}\right\},\;\;t=0,1,\ldots ,T-1.$$

Hence, for the SU, the energy causality constraint should satisfy

(8) $$\phi_{t}\delta +\theta_{t}E_{T,t}\leq b_{t},\;\;t=0,1,\ldots ,T-1.$$

(4) AoI model: We consider a linear model for the AoI [16], where the AoI is defined as the time elapsed from the moment when the most recently received update was generated to the present. Let the AoI at time slot *t* denote by $a_{t}\in A≜\left\{1,\;2,\;...,\;A_{\max}\right\}$. Here $A_{\max}$ is the upper of the AoI and is defined as

(9) $$A_{\max}=a_{0}+T.$$

In the considered system, the SU adopts the generate-at-will scheme. That is, the SU generates and delivers a status-update data pack after making an update decision. At each time slot *t*, the size of the data packet *S* is small enough to be generated and updated immediately and received by the end of the current time slot when the update decision is made and the sensing result $\hat{q_{t}}=I$ is correct. If the update is received at the CBS, AoI decreases to one; otherwise, it increases by one. We consider an error-free channel through which the status-update data pack can be successfully received at the CBS when the update decision is made and the sensing result $\hat{q_{t}}=I$ is correct. The average AoI for an interval of *T* time slots is expressed as

(10) $$\bar{A}=\frac{1}{T}\sum_{t=0}^{T-1}a_{t},\;\;t=0,1,\ldots ,T-1.$$

## 4. POMDP for AoI Minimization

In this section, we formulate the AoI minimization as a finite-horizon POMDP problem and solve for the optimal solutions via dynamic programming.

### 4.1. POMDP Formulation

We use a POMDP framework to model the optimal sensing and update decisions for the SU’s AoI minimization. The components of POMDP are described as follows.

- Actions: At the beginning of each time slot *t*, the SU needs to decide whether to sense the spectrum. If it decides not to sense the spectrum, then it captures energy from the PU transmissions and does not update, i.e., $x_{t}=\left(0,0\right)$. If it decides to sense the spectrum and finds that the PU is idle, it further decides whether to update based on the available energy, the AoI value, the channel state information from the SU to the CBS and from the PU to the SU, i.e., $x_{t}=\left(1,0\right)$ and $x_{t}=\left(1,1\right)$. Thus, the action for each time slot *t* is $x_{t}=\left(\phi_{t},\theta_{t}\right)\in X≜\left\{\left(0,0\right),\left(1,0\right),\left(1,1\right):b_{t}\geq \phi_{t}\delta +\theta_{t}E_{T,t}\right\}$, where $\phi_{t}\in \Gamma_{\phi }≜\left\{0,1:b_{t}\geq \phi_{t}\delta \right\}$ and $\theta_{t}\in \Gamma_{\theta }≜\left\{0,1:b_{t}\geq \delta +\theta_{t}E_{T,t}\right\}$.
- Observations and beliefs: Let ${\hat{q}}_{t}\in \left\{A,I\right\}$ denote the observation of the PU’s state. The belief $\rho_{t}\in \left[0,1\right]$ is a condition probability that the spectrum is vacated by the PU. The belief is updated according to the following cases.
  Case 1: The SU does not sense the spectrum; the new belief is given by
  (11) $$\rho_{t+1}=\Lambda_{0}\left(\rho_{t}\right)=\rho_{t}p_{ii}+\left(1-\rho_{t}\right)p_{ai}.\;$$
  Case 2: If the PU is sensed to be active, the SU harvests energy in the remaining time of the current time slot, i.e., the battery energy increases. This implies the true state of the PU is $q_{t}=A$. The belief is updated as
  (12) $$\rho_{t+1}=p_{ai}.\;$$
  Case 3: If the PU is sensed to be active, the SU does not harvest energy; i.e., the battery energy does not change and is lower than $B_{\max}$. This implies the true state of the PU is $q_{t}=I$. The new belief is expressed as
  (13) $$\rho_{t+1}=p_{ii}.\;$$
  Case 4: If the PU is sensed to be active, the battery energy is $B_{\max}$ at time slot *t*. The new belief is given by
  (14) $$\rho_{t+1}=\Lambda_{1A}\left(\rho_{t}\right)=\zeta_{t}p_{ii}+\left(1-\zeta_{t}\right)p_{ai},\;$$
  where
  (15) $$\zeta_{t}\;≜\;P\left(q_{t}\;=\;I|\hat{q_{t}}=A\right)=\frac{\rho_{t}\left(1-p_{f}\right)}{\rho_{t}p_{f}\;+\;\left(1-\rho_{t}\right)\left(1-p_{d}\right)}.\;$$
  Case 5: If the PU is sensed to be idle, the SU does not update. The belief is updated as
  (16) $$\rho_{t+1}=\Lambda_{1I}\left(\rho_{t}\right)=\bar{\zeta_{t}}p_{ii}+\left(1-\bar{\zeta_{t}}\right)p_{ai},\;$$
  where
  (17) $$\bar{\zeta_{t}}\;≜\;P\left(q_{t}\;=\;I|\hat{q_{t}}=I\right)=\frac{\rho_{t}\left(1-p_{f}\right)}{\rho_{t}\left(1-p_{f}\right)\;+\;\left(1-\rho_{t}\right)\left(1-p_{d}\right)}.\;$$
  Case 6: If the PU is sensed to be idle, the SU updates successfully. This implies that the true state of the PU is $q_{t}=I$. Then, we have
  (18) $$\rho_{t+1}=p_{ii}.\;$$
  Case 7: If the PU is sensed to be idle, the SU update fails. This implies that the true state of the PU is $q_{t}=A$. Then, we have
  (19) $$\rho_{t+1}=p_{ai}.\;$$
  Although (11)–(19) cover seven cases from case one to case seven, the new beliefs in both case two and case seven are denoted as $p_{ai}$, and the new beliefs in both case three and case six are denoted as $p_{ii}$. Hence, the SU can only transit to five beliefs. That is, the number of possible beliefs is finite over *T* time slots. Thus, for the length of *T* time slots, the belief space Φ is a finite set.
- States: Denote the discrete battery energy level of the SU at the beginning of time slot *t* by $b_{t}^{{}^{\prime }}\in B≜\left\{0,\;1,\;2,\;...,\;b_{\max}\right\}$, where $b_{\max}$ is the maximum battery energy level that can be stored inside the battery of the SU. Then, each energy quantum of the SU’s battery contains $\frac{B_{\max}}{b_{\max}}$ Joules. In this case, we use $b_{t}^{{}^{\prime }}=\left\lfloor \frac{b_{t}b_{\max}}{B_{\max}}\right\rfloor$ to convert the continuous battery energy of the SU to the discrete battery energy level, by which a lower bound to the AoI of the original continuous system is obtained. Similarly, divide continuous channel power gain into finite number of intervals according to fading probability density function (PDF). Thus, the discrete channel power gain levels from the SU to the CBS and from the PU to the SU are expressed as $g_{t}^{{}^{\prime }}\in G≜\left(0,\;1,\;2,\;...,\;g_{\max}\right)$ and $h_{t}^{{}^{\prime }}\in H≜\left(0,\;1,\;2,\;...,\;h_{\max}\right)$, respectively. Here, $g_{\max}$ and $h_{\max}$ denote the corresponding maximum channel power gain levels. At each time slot *t*, the completely observable states include channel state from the PU to the SU, channel state from the SU to the CBS, the AoI state, and battery state, denoted by $s_{t}≜\left(h_{t}^{{}^{\prime }},\;g_{t}^{{}^{\prime }},\;a_{t},\;b_{t}^{{}^{\prime }}\right)$. Note that the state space, i.e., S≜H×G×A×B, is finite. Due to imperfect sensing, an update may be unsuccessful when the sensing result is $\hat{q_{t}}=I$ and the update decision is $\theta_{t}=1$. Thus,
  (20) $$a_{t+1}\;\;=\;\;\left\{\begin{array}{cc} 1,\; & \mathrm{when}\;\;x_{t}=\left(1,1\right)\;\mathrm{and}\;\hat{q_{t}}=q_{t},\\ a_{t}+1,\; & \mathrm{otherwise}, \end{array}\right.$$
  for t=1,2,....,T. Alternatively, we can express $a_{t+1}=\left(1-\theta_{t}\right)a_{t}+1$ when the sensing result $\hat{q_{t}}=I$ is correct. Additionally, the PU’s spectrum state is only partially observable, which is described by the belief $\rho_{t}$. Thus, for each time slot *t*, the complete system state is denoted by $\left(s_{t},\rho_{t}\right)$. Since S and Φ are finite, the SU experiences a finite number of possible system states $(s_{t},\varrho_{t})\in S\times \Phi$.
- Transition probabilities: For time slot *t*, given the current state $s_{t}=\left(h_{t}^{{}^{\prime }},g_{t}^{{}^{\prime }},a_{t},b_{t}^{{}^{\prime }}\right)$ and the action $x_{t}=\left(\phi_{t},\theta_{t}\right)$, the transition probability to the next state $s_{t+1}=(h_{t+1}^{{}^{\prime }},g_{t+1}^{{}^{\prime }},a_{t+1},$$b_{t+1}^{{}^{\prime }})$ is denoted by $p_{x_{t}}\left(s_{t+1}|s_{t}\right)$. Since the captured energy and the channel power gains are independently and identically distributed (i.i.d), the transition probabilities for taking actions other than $x_{t}=\left(1,1\right)$ are given as follows.
  (21) $$\begin{array}{c} p_{x_{t}}\left(s_{t+1}|s_{t}\right)=P\left(a_{t+1}|a_{t},\;x_{t}\right)P\left(b_{t+1}^{{}^{\prime }}|b_{t}^{{}^{\prime }},\;g_{t}^{{}^{\prime }},\;h_{t}^{{}^{\prime }},\;x_{t}\right)P\left(g_{t+1}^{{}^{\prime }}\right)P\left(h_{t+1}^{{}^{\prime }}\right), \end{array}$$
  where
  (22) $$P\left(a_{t+1}|a_{t},\;x_{t}\right)\;\;=\;\;\left\{\begin{array}{cc} 1,\; & \mathrm{when}\;\;a_{t+1}=\left(1-\theta_{t}\right)a_{t}+1,\\ 0,\; & \mathrm{otherwise}, \end{array}\right.$$
  (23) $$\begin{array}{c} P\left(b_{t+1}^{{}^{\prime }}|b_{t}^{{}^{\prime }},\;g_{t}^{{}^{\prime }},\;h_{t}^{{}^{\prime }},\;x_{t}\right)=\left\{\begin{array}{cc} 1,\; & \mathrm{when}\;\;\phi_{t}=0\;\mathrm{and}\;b_{t+1}=\min\left\{\;b_{t}+E_{H,t}^{1},\;B_{\max}\right\},\\ 1,\; & \mathrm{when}\;\;\phi_{t}=0\;\mathrm{and}\;b_{t+1}=b_{t},\\ 1,\; & \mathrm{when}\;\;\phi_{t}=1,\;\theta_{t}=0,\;\mathrm{and}\;b_{t+1}=\min\left\{b_{t}-\delta +E_{H,t}^{2},\;B_{\max}\right\},\\ 1,\; & \mathrm{when}\;\;\phi_{t}=1,\;\theta_{t}=0,\;\mathrm{and}\;b_{t+1}=b_{t}-\delta ,\\ 0,\; & \mathrm{otherwise}. \end{array}\right. \end{array}$$
  For the action $x_{t}=\left(1,1\right)$, the transition probability is expressed as follows.
  (24) $$\begin{array}{c} p_{x_{t}}\left(s_{t+1}|s_{t},\hat{q_{t}},q_{t}\right)=P\left(a_{t+1}|a_{t},\;x_{t}\hat{q_{t}},q_{t}\right)\times P\left(b_{t+1}^{{}^{\prime }}|b_{t}^{{}^{\prime }},\;g_{t}^{{}^{\prime }},\;h_{t}^{{}^{\prime }},\;x_{t}\right)\times P\left(g_{t+1}^{{}^{\prime }}\right)P\left(h_{t+1}^{{}^{\prime }}\right), \end{array}$$
  where
  (25) $$P\left(a_{t+1}|a_{t},\;x_{t}\right)\;\;=\;\;\left\{\begin{array}{cc} \bar{\zeta },\; & \mathrm{when}\;\;a_{t+1}=1\;\mathrm{and}\;q_{t}=\hat{q_{t}},\\ 1-\bar{\zeta },\; & \mathrm{when}\;\;a_{t+1}=a_{t}+1\;\mathrm{and}\;q_{t}\leq \hat{q_{t}},\\ 0,\; & \mathrm{otherwise}, \end{array}\right.$$
  and
  (26) $$P\left(b_{t+1}^{{}^{\prime }}|b_{t}^{{}^{\prime }},\;g_{t}^{{}^{\prime }},\;h_{t}^{{}^{\prime }},\;x_{t}\right)=\left\{\begin{array}{cc} 1,\; & \mathrm{when}\;\;\phi_{t}=1,\;\theta_{t}=1,\;b_{t+1}=b_{t}-\delta -E_{T,t},\;\mathrm{and}\;\hat{q_{t}}=q_{t},\\ 1,\; & \mathrm{when}\;\;\phi_{t}=1,\;\theta_{t}=1,\;b_{t+1}=0,\;\hat{q_{t}}=I,\;\mathrm{and}\;q_{t}=A,\\ 0,\; & \mathrm{otherwise}. \end{array}\right.$$
- Cost: Let the immediate cost at state $s_{t}$ denoted by $C(s_{t})$, which is the accumulated AoI at time slot *t*. Then, we have
  (27) $$C\left(s_{t}\right)=a_{t},\;\;t=0,\;1,\;...,\;T-1.$$
- Policy: The policy is expressed as $\pi =\{\vartheta_{0},\;\vartheta_{1},\;...,\;\vartheta_{T-1}\}$, where $\vartheta_{t}$ is the deterministic decision rule that maps a system state $(s_{t},\rho_{t})\in S\times \Phi$ into an action $x_{t}\in X$, i.e., $x_{t}=\vartheta_{t}\left(s_{t},\rho_{t}\right)$. In this paper, let Π denote the set of all deterministic decision policies.

Given the SU’s initial state $s_{0}$ and belief $\rho_{0}$ of PU’s spectrum, the average AoI of *T* time slots under the policy π is given by

(28) $${\bar{A}}^{\pi }\left(s_{0},\;\rho_{0}\right)=\frac{1}{T}E\left[\sum_{t=0}^{T-1}C\left(s_{t}\right)|s_{0},\;\rho_{0}\right],$$

where the expectation is caused by policy π. Based on the above analysis, minimize the average AoI by finding the optimal sensing and update policy corresponds to solving

(29) $$\min_{\pi \in \Pi }\;{\bar{A}}^{\pi }\left(s_{0},\rho_{0}\right).$$

Given *T*, (29) is a finite-state MDP with total cost. Based on (28) and (29), to minimize the average AoI, the SU should sense the spectrum and deliver the status-update data pack as long as it has sufficient energy. However, considering the channel state information, the belief of PU’s spectrum, and the battery energy available, preferring the spectrum sensing and status-update may not be the best decision.As a result, there is an optimal decision scheduling problem.

### 4.2. POMDP Solution

In this section, we use dynamic programming to solve total cost minimization of *T* time slots in (29) [48]. At a time slot *t*, the successive actions ${\left\{x_{k}\right\}}_{k=t}^{T-1}$ affect the states $s_{k}$ along with the accumulated AoI $C(s_{k})$ for all k=t,t+1,…,T−1. Let $V_{t}\left(s_{t},\rho_{t}\right)$ denote the state-value function, which is given by

(30) $$V_{t}\left(s_{t},\rho_{t}\right)≜\min_{{\left\{x_{k}\right\}}_{k=t}^{T-1}}E\left[\sum_{k=t}^{T-1}C\left(s_{k}\right)|s_{t},\rho_{t}\right].$$

It is the minimum expected cost accumulated from time slot *t* to T−1 given state $\left(s_{t},\rho_{t}\right)$. Thus, denote the minimum AoI in (29) by $A^{*}=V_{0}\left(s_{0},\rho_{0}\right)/T$. Additionally, given $\left(s_{t},\rho_{t}\right)$ and sensing action $\phi_{t}$, let $Q_{t}^{\phi_{t}}\left(s_{t},\rho_{t}\right)$ represent the action-value function or Q-function, which is the minimum expected cost for taking sensing action $\phi_{t}$ at state $\left(s_{t},\;\rho_{t}\right)$. The Q-function includes two parts: the immediate cost of taking action at the current state and the expected sum of the state-value functions from the next time slot.

Overall, the formulated MDP problem can be solved recursively by dynamic programming as follows. For t=0,1,…,T−1,

(31) $$V_{t}\left(s_{t},\rho_{t}\right)=\min_{\phi_{t}\in \Gamma_{\phi }}Q_{t}^{\phi_{t}}\left(s_{t},\rho_{t}\right),$$

When t=T−1, we have

(32) $$\begin{array}{c} Q_{T-1}^{0}\left(s_{T-1},\rho_{T-1}\right)=C\left(s_{T-1}\right)+C\left(s_{T}\right), \end{array}$$

(33) $$\begin{array}{c} Q_{T-1}^{1}\left(s_{T-1},\rho_{T-1}\right)=\left(1-\Delta_{T-1}\right)C\left(s_{T-1}\right)+\rho_{T-1}\times \Delta_{T-1}\min_{\phi_{T-1}\in \Gamma_{\phi }}C\left(s_{T-1}\right)+C\left(s_{T}\right). \end{array}$$

When t=0,1,…,T−2, we have

(34) $$\begin{array}{c} Q_{t}^{0}\left(s_{t},\rho_{t}\right)=C\left(s_{t}\right)+\sum_{s_{t+1}}p_{00}\left(s_{t+1}|s_{t}\right)V_{t+1}\left(s_{t+1},\;\Lambda_{0}\left(\varrho_{t}\right)\right), \end{array}$$

(35) $$\begin{array}{c} Q_{t}^{1}\left(s_{t},\rho_{t}\right)=\left(1-\Delta_{t}\right)Q_{t}^{1A}\left(s_{t},\rho_{t}\right)+\Delta_{t}\min_{\theta_{t}\in \Gamma_{\phi }}Q_{t}^{1\phi_{t}}\left(s_{t},\rho_{t}\right), \end{array}$$

(36) $$\begin{array}{c} Q_{t}^{1A}\left(s_{t},\rho_{t}\right)=C\left(s_{t}\right)+\sum_{s_{t+1}}p_{10}\left(s_{t+1}|s_{t}\right)V_{t+1}\left(s_{t+1},\;\Lambda_{1A}\left(\varrho_{t}\right)\right), \end{array}$$

(37) $$\begin{array}{c} Q_{t}^{10}\left(s_{t},\varrho_{t}\right)=C\left(s_{t}\right)+\sum_{s_{t+1}}p_{10}\left(s_{t+1}|s_{t}\right)V_{t+1}\left(s_{t+1},\;\Lambda_{1I}\left(\varrho_{t}\right)\right), \end{array}$$

(38) $$\begin{array}{cc} Q_{t}^{11}\left(s_{t},\rho_{t}\right)= & C\left(s_{t}\right)\;+\;\sum_{s_{t+1}}p_{11}\left(s_{t+1}|s_{t},\;\hat{q_{t}}\;=\;q_{t}\right)V_{t+1}\left(s_{t+1},\;\Lambda_{I}\left(\varrho_{t}\right)\right)\\ \; & +\sum_{s_{t+1}}p_{11}\left(s_{t+1}|s_{t},\;\hat{q_{t}}\leq q_{t}\right)V_{t+1}\left(s_{t+1},\;\Lambda_{A}\left(\varrho_{t}\right)\right), \end{array}$$

where $\Delta_{t}$ represents the probability of observing PU idle. That is

(39) $$\Delta_{t}=P\left(\hat{q_{t}}=I\right)=\rho_{t}\left(1-p_{f}\right)+\left(1-\rho_{t}\right)\left(1-p_{d}\right).$$

Especially, $Q_{t}^{1A}\left(s_{t},\rho_{t}\right)$ in (36) represents the minimum expected cost by adopting sensing action $\phi_{t}=1$ and sensing result ${\hat{q}}_{t}=A$, i.e., $x_{t}=\left(1,0\right)$. In (37) and (38), given the sensing action $\phi_{t}=1$ and sensing result ${\hat{q}}_{t}=I$, $Q_{t}^{10}\left(s_{t},\varrho_{t}\right)$ and $Q_{t}^{11}\left(s_{t},\varrho_{t}\right)$ denote the minimum expected costs by adopting update action $\theta_{t}=0$ and $\theta_{t}=1$, respectively. Then, by recursion in (31)–(38), the optimal policies for sensing and update are given by

(40) $$\begin{array}{c} \phi_{t}^{*}\left(s_{t},\rho_{t}\right)\in \underset{\phi_{t}\in \Gamma_{\phi }}{\mathrm{argmin}}\;Q_{t}^{\phi_{t}}\left(s_{t},\varrho_{t}\right), \end{array}$$

(41) $$\begin{array}{c} \theta_{t}^{*}\left(s_{t},\rho_{t}\right)\in \underset{\phi_{t}\in \Gamma_{\theta }}{\mathrm{argmin}}\;Q_{t}^{1\theta_{t}}\left(s_{t},\varrho_{t}\right). \end{array}$$

## 5. Numerical Results

In this section, we evaluate the performance of our proposed optimal policy through comparing it with the myopic policy and the random policy. At the beginning of time slot *t*, for the myopic policy, the SU senses the spectrum if it has enough energy. When the sensing result is $\hat{q_{t}}=I$, the SU generates and delivers a status-update data pack if the energy available is sufficient. For the random policy, the SU randomly chooses to deliver the status-update data pack or harvest energy with a probability. Taking into account the protection of the PU’s transmission, the probability of harvesting energy is set to be 90%, and the probability of delivering the status-update data pack is set to be 10%. If the SU chooses to deliver the status-update data pack while the spectrum is occupied by the PU, the status-update fails, and the AoI increases by one. The PU’s state transition probabilities are $p_{ii}=0.8$ and $p_{ai}=0.5$. The probability of detecting an active PU is $p_{d}=0.8$. The channel power gains from the PU to the SU and from the SU to the CBS are modeled as $h=Y\Psi^{2}d_{1}^{-\kappa }$ and $g=Y\Psi^{2}d_{2}^{-\kappa }$, where $d_{1}$ and $d_{2}$ denote the distances from the PU to the SU, and the SU to the CBS, respectively. Y represents a signal power gain at a 1 m’s reference distance, Ψ∼exp(1) denotes the small-scale fading gain, and $d_{1}^{-\kappa }$ and $d_{2}^{-\kappa }$ are standard power law path-loss with exponent κ. In the simulations, the system parameter values are set as follows: η=0.5, $\sigma^{2}=-95$ dBm, W=1 MHz, Y=0.2, κ=2, $b_{\max}=5$, $g_{\max}=h_{\max}=10$, $\rho_{0}=p_{ii}$, $\tau_{s}=0.2$ s, and $A_{\max}=13$.

**Figure 2:**
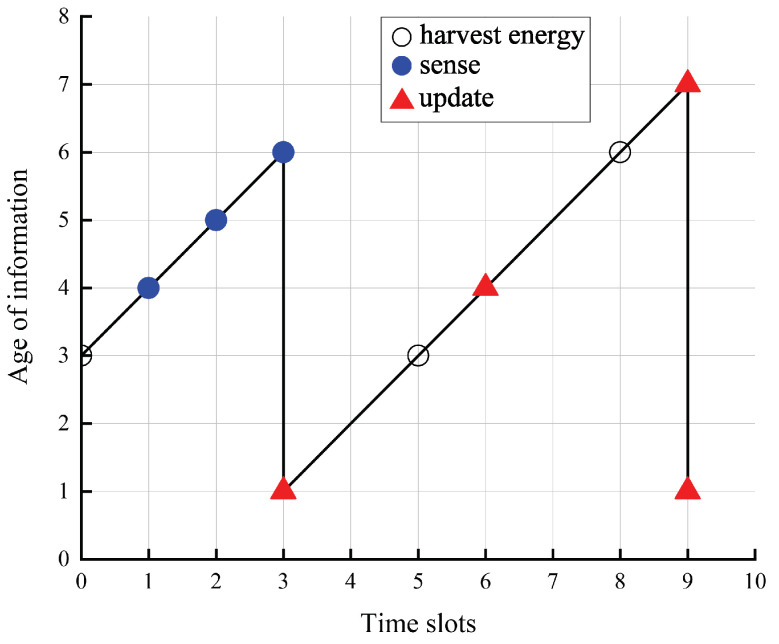
One sample path of the AoI by the optimal policy.

Figure 2 shows one sample path of the AoI by the optimal policy. The transmit power of the PU is 35 dBm, the energy consumption is one energy quantum, the distance from the the SU to the CBS is 20 m, the distance from the PU to the SU is 25 m, the size of the status-update data pack is 14 Mbits, and the battery capacity is 0.5 mJoules. The trend of the AoI over time slots is clearly observed. In the simulations, we found the SU did not perform sensing spectrum even the remaining energy was enough, which verifies the foresight of the optimal policy compared with the myopic policy.

**Figure 3:**
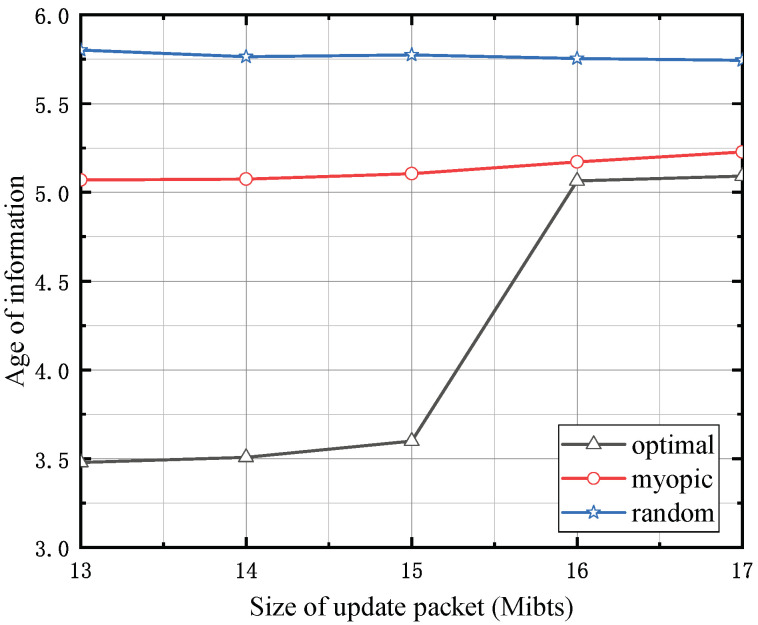
The size of status-update data packet versus the AoI when *T* = 10.

Figure 3 shows the size of the status-update data packet versus the AoI, where the simulation setup is similar as in Figure 3. It is clear that our proposed policy is superior to the other policies. For the random policy, the AoI is obviously high due to the low probability of delivering the status-update data pack. For the random policy, the AoI is greater than 5.57, due to the low probability of delivering the status-update data pack. Considering the poor AoI performance of the random policy, we only compare our algorithm with the myopic algorithm in the following numerical evaluations. We can observe that the AoI increases with the size of the status-update data packet. The reason is that the increase in the size of the status-update data packet will result in increasing the energy needed to deliver one status-update data pack. This decreases the possibility that the SU will have enough energy to update, and hence the AoI is increased.

**Figure 4:**
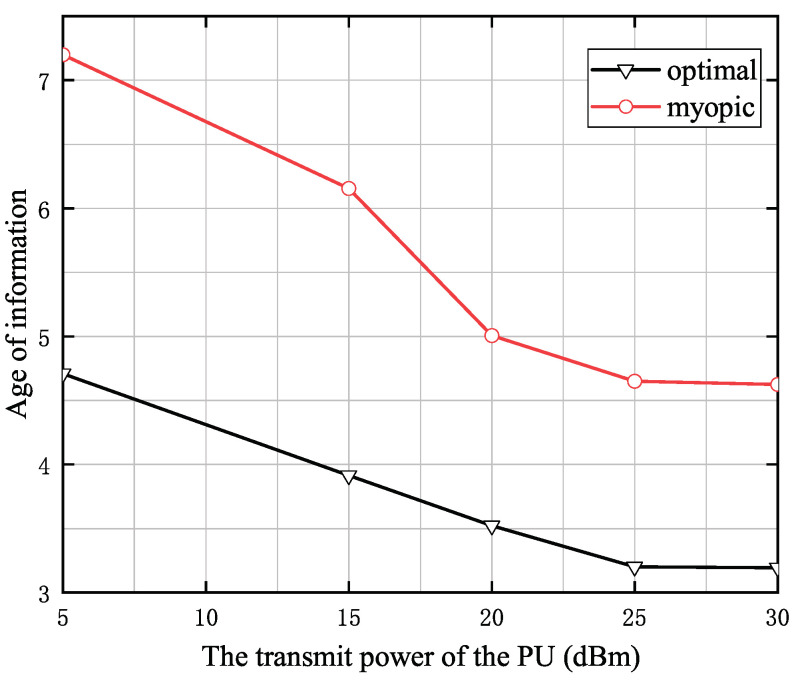
The transmit power of PU versus the AoI when *T* = 10.

Figure 4 shows the transmit power of the PU versus the AoI, where the capacity of battery is 0.2 mJoules, the distance from the PU to the SU is 5 m, the distance from SU to the CBS is 25 m, the size of status-update data pack is 15 Mbits. We can observe from Figure 4 that the average AoI increases with the transmit power of PU. The reason is that the SU will harvest more energy as the transmit power of PU increases, which allows the SU to store more energy in the battery. This increases the possibility that the SU will have enough energy to update, and hence the AoI is decreased.

**Figure 5:**
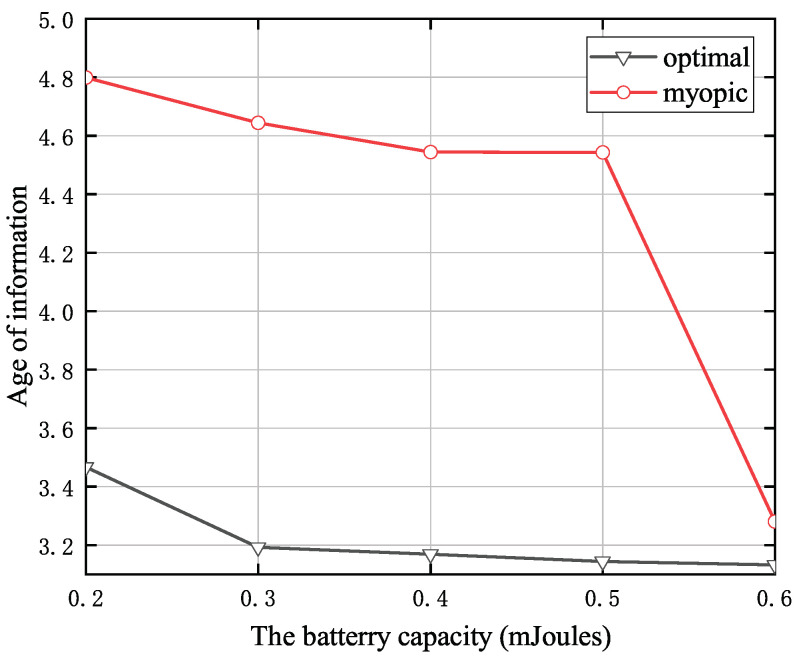
The battery capacity versus the AoI when *T* = 10.

Figure 5 shows the battery capacity versus the AoI, where the size of the status-update data pack is 15 Mbits, the energy consumption on the sensing spectrum is one energy quantum, the transmit power of the PU is 35 dBm, the distance from the SU to the CBS is 10 m, and the distance from the PU to the SU is 5 m. It is clearly observed that the proposed policy essentially improves the AoI as compared to the myopic policy. We can also observe the average AoI decreases with the battery capacity. The reason is that increasing the battery capacity allows more harvested energy to be stored inside the battery. Thus, the SU will have enough energy to perform an update, and hence the AoI is reduced.

**Figure 6:**
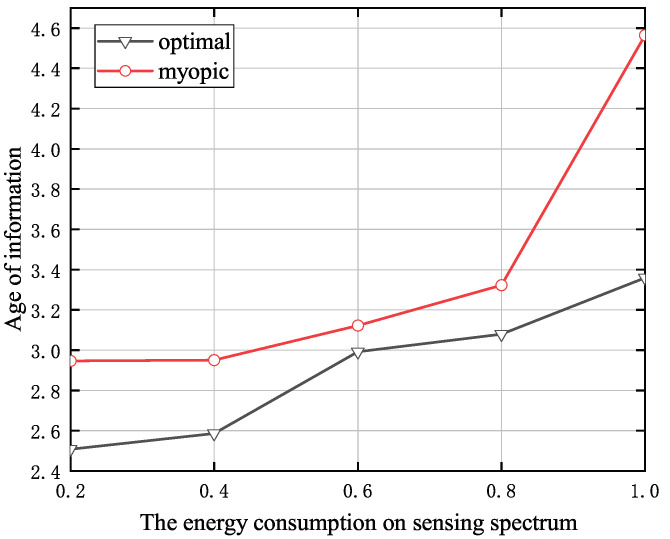
The energy consumption on sensing spectrum versus the AoI when *T* = 10.

Figure 6 shows the energy consumption on sensing spectrum versus the AoI. The simulation setup is the similar as in the Figure 5. It is observed that the average AoI increases with the energy consumption on sensing action. The reason is that increasing the energy consumption on sensing spectrum can result in less energy remaining inside the battery. This, in turn, decreases the possibility that the SU will have enough energy to deliver status-update data packet, and hence the AoI is increased.

## 6. Conclusions

In this paper, we investigated RF energy-harvesting CRN with the aim of AoI minimization subject to the energy causality and spectrum constraints. We first used POMDP to formulate this average AoI minimization based on the AoI value, the channel state information, the energy available, and the PU’s spectrum belief, and then dynamic programming was adopted to find the optimal sensing and update decisions. Numerical results showed the influence of system parameters on the AoI, and demonstrated that the proposed policy significantly outperform the myopic policy.

## Figures and Tables

**Table 1:** List of notations used in this paper.

Notation	Definition
$p_{ai}$	The transition probability from the active state to the idle state
$p_{ii}$	The transition probability from the idle state to the idle state
$p_{f}$	The false alarm probability
$p_{d}$	The detection probability
$\phi_{t}$	The sensing decision at time slot *t*
$\theta_{t}$	The update decision at time slot *t*
$\hat{q_{t}}$	The sensing result
δ	The energy consumption on sensing spectrum
$\tau_{s}$	The time consumption on sensing spectrum
$E_{T,t}$	The energy consumption on update
$\tau_{t}$	The time consumption on update
*S*	The size of status-update data pack
$a_{t}$	The AoI at time slot *t*
$\rho_{t}$	The belief probability
$b_{max}$	The maximum battery energy level
$g_{\max}$	The maximum channel power gain level from the SU to the CBS
$h_{\max}$	The maximum channel power gain level from the PU to the SU
Φ	The belief space
η	The energy-harvesting efficiency
$\sigma_{2}$	The noise power
$B_{\max}$	The battery capacity of the SU
$A_{\max}$	The upper of AoI
$s_{t}$	The current state
$x_{t}$	The action at time slot *t*

## Data Availability

Not applicable.
